# Leukocytosis and high hematocrit levels during abdominal attacks of hereditary angioedema

**DOI:** 10.1186/1471-230X-13-123

**Published:** 2013-08-02

**Authors:** Isao Ohsawa, Seiji Nagamachi, Hiyori Suzuki, Daisuke Honda, Nobuyuki Sato, Hiroyuki Ohi, Satoshi Horikoshi, Yasuhiko Tomino

**Affiliations:** 1 Division of Nephrology, Department of Internal Medicine, Juntendo University Faculty of Medicine, Tokyo, Japan; 2 Internal Medicine, Tsurumi-Nishiguchi Hospital, Kanagawa, Japan

**Keywords:** Hereditary angioedema, C1-inhibitor, C1-inhibitor concentrate, Acute abdomen, Leukocytosis, Hemoconcentration

## Abstract

**Background:**

The diagnosis of hereditary angioedema (HAE) is often delayed due to the low awareness of this condition. In patients with undiagnosed HAE, abdominal symptoms often create the risk of unnecessary surgical operation and/or drug therapy. To explore the cause of misdiagnosis, we compared the laboratory findings of HAE patients under normal conditions with those during abdominal attacks.

**Methods:**

Patient medical histories were analyzed and laboratory data at the first consultation with no symptoms and no medication were compared with those at visits to the emergency department during severe attacks.

**Results:**

Fourteen HAE patients were enrolled. Initial HAE symptoms occurred at 20.2 ± 9.4 years of age. The correct diagnosis of HAE was made 22.7 ± 14.2 years after the initial symptoms. A common site of angioedema was the extremities. Half of the patients experienced a life-threatening laryngeal attack and/or severe abdominal pain. In the patients with severe abdominal pain, significant leukocytosis with neutrophilia along with increased levels of hematocrit were observed while levels of C-reactive protein (CRP) remained low. All severe attacks were alleviated with an infusion of C1-inhibitor concentrate.

**Conclusions:**

Consideration of the likelihood of a HAE attack is important when patients present with acute abdominal pain and leukocytosis without elevation of CRP.

## Background

Hereditary angioedema (HAE) is an autosomal dominant disease, caused by an inherited deficiency of functionally active C1 inhibitor (C1-INH). Episodic local angioedema is due to the release of bradykinin from high molecular weight kininogen via activation of the contact system which increases vascular permeability during HAE attacks [1]. Clinically, HAE is characterized by the presence of subcutaneous or submucosal tissue swelling, usually affecting the face, extremities, upper airways and/or gastrointestinal tract (GI-tract) [2].

While HAE is known to be inherited in an autosomal dominant form, 20**-**30% of cases develop as a result of *de novo* mutation [3]. The prevalence of this disease has been estimated at 1 case per 50,000 people, with no reported bias in different ethnic groups [1,3,4]. Plasma-derived C1-INH concentrate has been the first-line treatment for acute HAE attacks for decades, however, new drugs, such as a bradykinin B2 receptor antagonist and a kallikrein inhibitor are available in some countries [2]. Despite the availability of treatments for HAE, the global disease recognition rate is low. We previously conducted a recognition survey of HAE among Japanese physicians: 55% of all responding physicians (n = 4,495) failed to recognize HAE and 11% overlooked or misdiagnosed their patients [5]. Recently, we established a HAE Information Center on the Internet [6] (in Japanese), which aims to promote the recognition of HAE.

When severe edema develops in the airways, a patient’s condition may become life-threatening. When the GI-tract is involved in a HAE attack, edema of the gastrointestinal walls causes abdominal pain, nausea, vomiting and intestinal obstruction which is sometimes similar to that of an acute abdomen and is difficult to distinguish from a surgical emergency [7]. To date, no detailed observations are available which describe the laboratory data in HAE patients having abdominal attacks. In this study, HAE patients were followed and the laboratory findings of patients with no symptoms versus those with critical abdominal attacks were retrospectively compared.

## Methods

At the outpatient clinic of Juntendo University Hospital, Tokyo, Japan, HAE patients were followed from January 2011 to August 2012. They were diagnosed based on their clinical histories and laboratory data (antigenic levels of C4 and functional levels of C1-INH). As the evaluation of the antigenic level of C1-INH is not approved by the Japanese health insurance system, the HAE diagnosis criteria described by Agostoni et al. [8] was used for these patients. The study was conducted in accordance with the Declaration of Helsinki (1964) and was approved by the Institutional Review Board at Juntendo University. Written informed consent was obtained from all participating patients.

Family and past medical histories were collected from patients’ medical records. Blood samples were obtained after each patient’s first consultation for differential diagnosis of angioedema under normal conditions (no swelling or medication). All blood samples were obtained from the antecubital vein and biochemical analyses, including white blood cells (WBC), hemoglobin, hematocrit (Hct), serum creatinine, total protein, albumin, immunoglobulin (Ig) G, IgA, IgM, C3, C4, total hemolytic complement (CH50), C-reactive protein (CRP), anti-nuclear antibody (ANA), and immune complex (C1q-binding assay) were performed. Functional levels of C1-INH were determined using a chromogenic assay (Sysmex, Hyogo, Japan). Blood was donated by 10 of 14 patients for determining the antigenic level of C1-INH which was evaluated using nepherometry with antisera to C1-INH (Dade Behring, Inc., Illinois, America). When patients presented at the emergency department with a severe attack, the following laboratory data were re-evaluated: blood counts, WBC, C3, C4, CH50 and CRP in sera. Abdominal computed tomography (A-CT) and ultrasonography were performed as needed. Based on the physicians’ decision, 20 U/kg bodyweight (bw) C1-INH concentrate (Berinert® P, CSL Behring K.K.) was administered intravenously. No patients received prophylactic treatment with drugs such as androgens, kallikrein inhibitor and bradykinin B2 receptor antagonist throughout their clinical passage.

The GraphPad Prism 5J software for Windows (version 5.04; GraphPad, San Diego, CA, USA) was used for statistical analysis, and the two-sided p-value < 0.05 was taken as the level for statistical significance. All data were expressed as mean ± standard deviation (SD).

## Results

Fourteen patients with HAE (5 men and 9 women) visited our outpatient clinic. The mean (±SD) age was 44.1 ± 14.4 years. Initial HAE symptoms occurred at 20.2 ± 9.4 years of age, with a delay in correct diagnosis of HAE of 22.7 ± 14.2 years after the initial symptoms. A family history of angioedema was noted in 12 of the 14 patients (86%). All of the patients had experienced self-limiting, noninflammatory subcutaneous angioedema without major urticarial rash, often recurrent and often lasting for more than 12 hours. Trauma, dental procedure and stress could have been the triggering episode, but many attacks occurred without an identifiable stimulus. The frequencies of episodic swelling varied between patients. Swelling of extremities was a common symptom but the affected organs varied by patient. Almost all attacks spontaneously resolved within one hour to three days, however, six patients (No. 1, 6, 7, 8, 9, and 12) experienced a recurrent life-threatening laryngeal attack and/or severe abdominal pain. These severe symptoms had been misinterpreted as allergies, asthma, appendicitis, GI-tract perforation, GI-tract bleeding and/or enterocolitis. Two patients (No. 1 and 6) had previously undergone tracheotomy because of airway obstruction. Three patients had received abdominal surgery due to appendicitis, an ovarian cyst and myoma.

Table 1 shows laboratory data of results obtained on the first visit to our outpatient clinic (when there were no symptoms). Serum levels of C3 were within or above the normal range (69-128 mg/dL) in all but one patient (No. 14) while levels of C4 were below the normal range (14-36 mg/dL) in all cases. Functional levels of C1-INH were also below the normal range (70-130%) in all patients. Therefore, all patients fulfilled the criteria for the diagnosis of HAE [8]. One patient (No.11) had a weakly positive ANA titer (1:80) while all others were negative. Four patients (No. 2, 4, 9, and 10) gave positive results for immune complex, but their first symptoms of angioedema had occurred before the age of 15 years and they had no clinical symptoms of autoimmune diseases.

**Table 1:** Laboratory data of all hereditary angioedema (HAE) patients (at first visit without symptoms)

**Patient no.**	**C3 (mg/dL)**	**C4 (mg/dL)**	**CH5O (U/mL)**	**Cl-INH**		**WBC (/μL)**	**RBC (×10**^**4**^**/L)**	**Htc (%)**	**CRP (mg/dL)**
				**Function (%)**	**Antigen (mg/dl)**				
Normal range	69-128	14-36	25-54	70-130	11-26	3600-8900	380-504	35.6-45.4	<0.2
1	159	3	24.2	35	n.t.	7100	512	42.2	2.1
2	103	<2	9.7	<25	7	5100	414	39.1	0.3
3	100	9	39	30	<6	4100	487	43.8	0.2
4	140	5	33.4	29	10	4900	430	39.2	0.7
5	93	3	22.9	<25	n.t.	5900	475	43.6	0.1
6	106	7	33	29	8	7800	454	40.7	0.2
7	105	3	27	<25	<6	7700	490	44.7	0.4
8	n.t.	n.t.	n.t.	n.t.	n.t.	n.t.	n.t.	n.t.	n.t.
9	97	4	25.8	<25	7	6100	462	39.9	0.2
10	97	5	25.1	<25	<6	8400	470	43.2	0.1
11	112	10	51.5	39	14	9700	432	41.6	0.1
12	124	8	46	<25	n.t.	7000	526	47.1	0.1
13	95	9	33	33	10	3800	470	43.2	0.0
14	67	10	32.3	34	15	5600	365	36.1	0.0

*C1-INH* C1 inhibitor, *CH50* total hemolytic complement; *CRP* C-reactive protein, *Hct* hematocrit, *n.t.* not tested, *RBC* red blood cell, *WBC* white blood cell.

A total of 31 visits to the emergency department were recorded (Table 2). Of these, 28 were due to severe attacks and the patients were treated with C1-INH concentrate (20 U/kg bw). C1-INH concentrate therapy was effective in alleviating symptoms and all patients returned home without hospital admission. Relatively mild attacks (foot and lips) either received no medication or were treated with tranexamic acid.

**Table 2:** Total frequencies of emergency department visits, affected organs and treatment

**Total frequency of visits**	**31**	
C1-inhibitors concentrate	28	
GI tract	17	
GI tract		16
GI tract + Neck		1
Others	11	
Lips		3
Extremities		3
Cheek		2
Face		1
Face + Neck + Hand		1
Shoulder + Larynx		1
Tranexamic acid	1	
Foot		1
Observation (No medication)	2	
Lips		1
Foot		1

*GI-tract* gastrointestinal tract.

**Figure 1:**
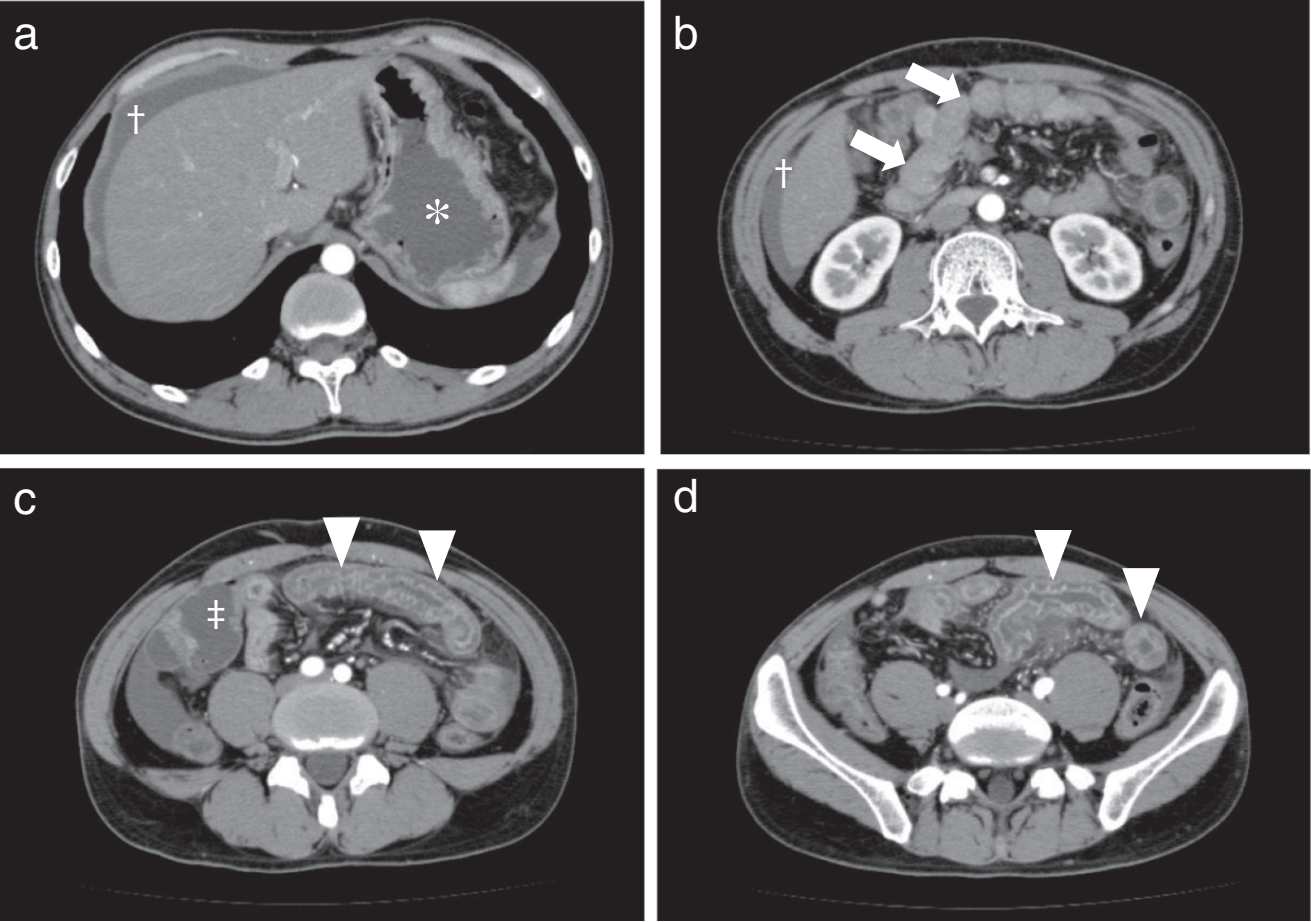
**Enhanced abdominal computed tomography (A-CT) of severe gastrointestinal edema. a**: Liver-stomach level slice, **b**: Kidney level slice, **c**: Under umbilical level slice, **d**: Pelvis level slice. Patient No. 3 visited the emergency department because of severe abdominal pain and vomiting. Obstructive duodenum (arrow) of the small intestine (arrow head) was visualized due to the thickening of the gastrointestinal walls. Gastric (*) and intestinal walls (‡) were expanded. Moderate ascites was observed around liver (†). (Photos were provided by Dr. N. Hosoi, Tokyo-Kita social insurance hospital).

Severe colicky abdominal pain was observed a total of 17 times in six of 14 patients. Figure 1(a-d) shows representative A-CT images of a patient (aged 33 years, male) with thickening of the gastrointestinal wall and ascites. The stomach was distended due to a GI-tract obstruction.

**Figure 2:**
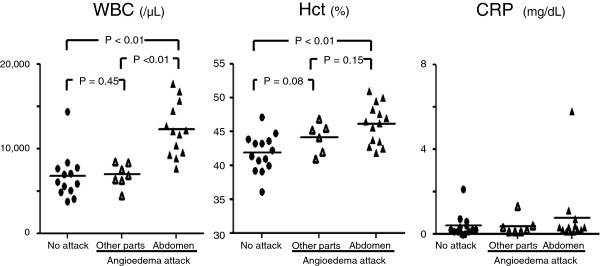
Measurement of white blood cells (WBC), hematocrit (Hct) and C-reactive protein (CRP).

**Figure 3:**
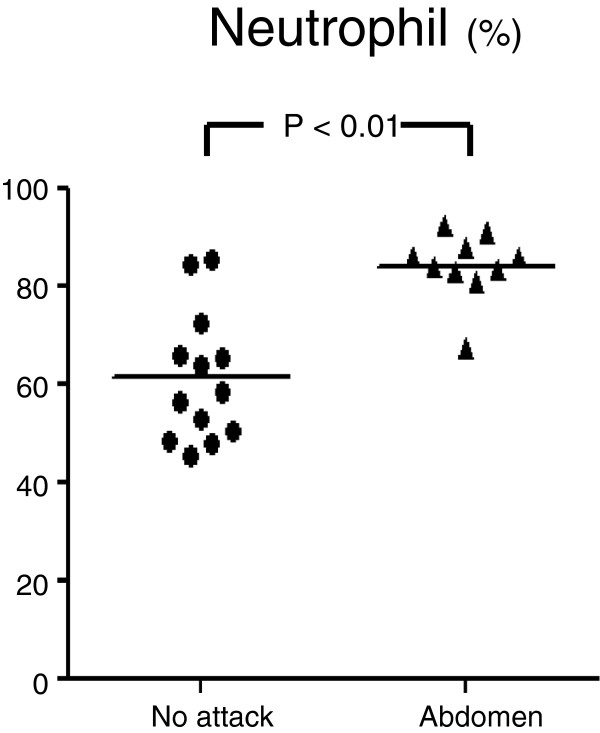
Percentage of neutrophils in the white blood cells (WBC) of the peripheral blood.

The number of WBCs at the time of severe abdominal attack was higher (12,253 ± 3,148 /μL) than those under normal conditions (6,400 ± 1,740 /μL) [p < 0.01] and during attacks involving other sites (6,942 ± 1,393 /μL) [p < 0.01]. However, the levels of CRP were low during normal conditions, abdominal attacks and attacks at other sites (Figure 2). The number of red blood cells at the time of severe abdominal attack (527 ± 50 ×10^4^/μL) was higher than that under normal conditions (459 ± 42×10^4^/μL) [p < 0.01]. The level of Hct at the time of severe abdominal attack (46.1 ± 3.0%) was higher than that under normal conditions (41.7 ± 2.8%) [p < 0.01]. The percentage of neutrophils was higher at the time of severe abdominal attack compared with that under normal conditions (84.1 ± 6.9% versus 61.2 ± 13.2%, respectively; [p < 0.01]) [Figure 3].

## Discussion

While the actual diagnosis of HAE is commonly made in the second or third decade of life, symptoms often begin earlier. As the symptoms of HAE mimic other disorders they are often misinterpreted [8]. An international consensus algorithm for the diagnosis of HAE [9] and a Japanese guideline of HAE [10] have been published and describe the determination of antigenic levels of C1-INH as needed for the classification of HAE into type I or type II. However, Japanese health insurance limitations have created a problem in this evaluation. In our study, taking a detailed history of the recurrent angioedema as well as a family history was helpful for suspecting HAE. The detection of low levels of C4 is the key test for screening HAE patients. Our examination confirmed the diagnosis of HAE in 100% of patients by measuring functional levels of C1-INH and serum levels of C4 (normal levels of C4 virtually exclude HAE) [9,11,12]. Although a small number of patients were enrolled in this study, the gender ratio and number of patients with a family history of angioedema were comparable with previous reports [1,3,9].

Frank et al. reported that abdominal pain, nausea and vomiting are the dominant symptoms of HAE in approximately 70-80% of all patients and typically ameliorate within five days [13]. Furthermore, approximately one-third of patients with abdominal attacks undergo unnecessary surgery, such as appendectomy and exploratory laparotomy [14]. Since these abdominal symptoms are relatively nonspecific, the response to treatment with C1-INH concentrate may be the only way to differentiate a HAE attack from a surgical condition [15].

In this study patients visited the emergency department 31 times: 28 of the 31 attacks were severe and treated with C1-INH concentrate which is the only licensed HAE therapy in Japan. After treatment, all symptoms resolved without any side effects and all patients were able to return home.

To date, there has only been one case report describing the occurrence of leukocytosis during an abdominal HAE attack [16]. In our study, the number of WBCs in patients with abdominal attacks was significantly higher than those under normal conditions and during attacks at other sites, while serum levels of CRP showed no significant difference. Although no statistical correlation was observed between attack duration and the number of WBCs, in all cases where an attack had started over 8 hours before the emergency department visit, the number of WBCs was over 12,000 μL. In the context of a usual bacterial infection, leukocytosis is recognized prior to an increase of CRP. Such a situation mimics the super-acute state of a GI-tract infection (e.g., appendicitis) and/or gastrointestinal perforation; however, our patients’ symptoms were alleviated without antibiotics. The main population of WBCs showed increased neutrophils without an increase of CRP. We have not addressed the mechanisim of alteration of the WBC population, although it is possible to envisage a scenario in which these findings demonstrate an unexpected role for the sympathetic nervous system. The number of neutrophils is known to be altered in the response to non-pathologic situations such as emotional or physical stressors [17]. These mechanisms are explained by the β-adrenergic effect of epinephrine, which diminishes neutrophil adherence to the endothelial cells [18,19].

When HAE patients presented with severe abdominal pain, we also found a significant increase in Hct. Simultaneously, the levels of total protein and albumin in sera tended to increase during abdominal attacks (data not shown) but lacked significance. Since red blood cells cannot pass through capillary walls, we could obtain a statistically significant difference. Our previous report also presented a case of severe angioedema which had spread to the patient’s whole body, contributing to a weight gain of nearly 20 kg [20]. It appears that dehydration and the fluctuation of Hct reflects massive fluid translocation from the blood stream to the GI-tract wall. If angioedema could not be alleviated, these severe cases would register hypovolemic shock from extravascular fluid loss [21] and mimic GI-tract bleeding or vagotonic shock due to intolerable pain.

## Conclusions

During abdominal attacks in patients with HAE, leukocytosis and high Hct without CRP elevation can be confused with an acute abdomen needing an emergency surgical procedure. Thus, consideration of the likelihood of a HAE attack is of clinical importance in such cases.

## Competing interests

None of the authors have any financial or non-financial competing interests influencing the interpretation of data or presentation of information.

## Authors’ contributions

IO followed up patients and carried out the study. SN, HS and DH performed the experiments and helped to draft the manuscript. IO and NS designed the study and carried out the statistical analysis. HO, SH and YT revised the manuscript. YT is the corresponding author. All authors read and approved the final manuscript.

## Pre-publication history

The pre-publication history for this paper can be accessed here:

http://www.biomedcentral.com/1471-230X/13/123/prepub
